# Blacktip reef sharks (*Carcharhinus melanopterus*) show high capacity for wound healing and recovery following injury

**DOI:** 10.1093/conphys/cov062

**Published:** 2015-12-21

**Authors:** Andrew Chin, Johann Mourier, Jodie L Rummer

**Affiliations:** af1 Centre for Sustainable Tropical Fisheries and Aquaculture & College of Marine and Environmental Sciences, James Cook University, Townsville, QLD 4811, Australia; af2 Laboratoire d'Excellence 'CORAIL, USR 3278 CRIOBE CNRS-EPHE-UPVD, CRIOBE BP 1013 Moorea, 98729 Polynésie française; af4 ARC Centre of Excellence for Coral Reef Studies, James Cook University, Townsville, QLD 4811, Australia

**Keywords:** Coral reefs, elasmobranchs, fish, fisheries, French Polynesia, Great Barrier Reef

## Abstract

Wound healing is important for sharks from the earliest life stages, for example, as the ‘umbilical scar’ in viviparous species heals, and throughout adulthood, when sharks can incur a range of external injuries from natural and anthropogenic sources. Despite anecdotal accounts of rapid healing in elasmobranchs, data regarding recovery and survival of individuals from different wound or injury types has not been systematically collected. The present study documented: (i) ‘umbilical scar’ healing in wild-caught, neonatal blacktip reef sharks while being reared for 30 days in flow-through laboratory aquaria in French Polynesia; (ii) survival and recovery of free-swimming blacktip reef sharks in Australia and French Polynesia following a range of injuries; and (iii) long-term survival following suspected shark-finning activities. Laboratory monitoring, tag-recapture records, telemetry data and photo-identification records suggest that blacktip reef sharks have a high capacity to survive and recover from small or even large and severe wounds. Healing rates, recovery and survival are important factors to consider when assessing impacts of habitat degradation and fishing stress on shark populations. The present study suggests that individual survival may depend more on handling practices and physiological stress rather than the extent of physical injury. These observations also contribute to discussions regarding the ethics of tagging practices used in elasmobranch research and provide baseline healing rates that may increase the accuracy in estimating reproductive timing inferred from mating scars and birth dates for neonatal sharks based on umbilical scar healing status.

## Teaser

Wounds heal quickly in blacktip reef sharks. In captive neonates, umbilical scars heal within weeks. In mature individuals, predator wounds and/or telemetry tag surgical procedure wounds heal in weeks to months. This information is important for monitoring populations and assessing habitat degradation impacts and interactions with fishing gear on sharks.

## Introduction

Sharks and rays incur injuries throughout all life stages that originate from many sources, including mating and courtship behaviours, parturition (for females and neonates), aggression, predation and human activities (Carrier *et al.*, 1994; Towner *et al.*, 2012; Hoyos-Padilla *et al.*, 2013). However, elasmobranchs are reported to be naturally resilient to some types of injuries. For example, in some species the skin is up to twice as thick in females compared with males and is thought to protect the females during aggressive mating behaviours (Pratt, 1979; Kajiura *et al.*, 2000). Large sharks have also exhibited recovery from penetrative harpoon wounds (Riley *et al.*, 2009) and abrasion caused by marine debris (Wegner and Cartamil, 2012). For these reasons and based on several studies, it is suggested that tagging with external fin tags does not endanger sharks and rays (Heupel *et al.*, 1998). Nevertheless, with the exception of anecdotal evidence, there are relatively few records that document healing and recovery from the range of wounds experienced by sharks. Furthermore, such information is not only important to the welfare of individuals and sustainability of populations, but mating and umbilical scars, for example, can also be used to indicate timing of reproduction in elasmobranchs (Walker, 2005). However, healing rates are neither well documented nor understood, and therefore there is uncertainty in the use of mating or umbilical scars to predict the timing of mating or birthing events. Concerns have also been raised about the health and well-being of sharks after tagging, and there is an ongoing debate about the ethics of tagging procedures used in acoustic monitoring, a rapidly growing research area (Heupel and Webber, 2012). However, there are few explicit accounts of healing rates and survival after internal insertion of acoustic tags to inform this discussion.

Although it has been reported that sharks have high capacity to heal and recover from wounds, there are few published data on healing rates and recovery, and very few data on healing from a range of injury types in the same species thorughout early life stages into adulthood. The blacktip reef shark (*Carcharhinus melanopterus*) is a widespread and abundant predator across the Indo-Pacific (Vignaud *et al.*, 2014) that is adapted to a diverse range of conditions from shallow-water lagoons (Papastamatiou *et al.*, 2009) to outer reefs (Mourier *et al.*, 2012) and turbid, hypoxic waters of inshore bays (Chin *et al.*, 2013c). This medium-sized reef shark is a good candidate to address questions about healing and recovery, not only because of their distribution, but also because they show evidence of aggressive mating activity (McCauley *et al.*, 2010), and mature females can be observed with obvious mating scars (Porcher, 2005; Chin *et al.*, 2013b). Blacktip reef sharks may also incur injuries from aggressive behaviour and predation by larger reef sharks, such as the grey reef shark (*Carcharhinus amblyrhynchos*) and sicklefin lemon shark (*Negaprion acutidens*), and potentially, by very large sharks, such as the great hammerhead shark (*Sphyrna mokarran*), which has been observed to prey upon reef sharks (Mourier *et al.*, 2013b). Here, we provide photographic records and quantitative data documenting the recovery of blacktip reef sharks from natal umbilical scars and from injuries observed in free-swimming animals and from acoustic tagging data. These data will help to provide a greater understanding of the potential for recovery and survival from natural and anthropogenic injuries in this species and for elasmobranchs in general.

## Methods and materials

### Umbilical scars of neonatal sharks in Moorea

We collected eight neonate blacktip reef sharks (*C. melanopterus*) from shallow lagoons around the north coast of Moorea, French Polynesia using gill nets (50 m long, 1.5 m high, 6 cm mesh) set with fresh bait in 2014 during known pupping months (October–November; Mourier *et al.*, 2013a; Mourier and Planes, 2013). Upon collection, all sharks were measured (total length, fork length and body mass), sexed, and tagged in their dorsal fin, using T-bar coloured anchor tags (Hall-print, Hindmarsh Valley, SA, Australia) to allow sharks to be identified visually in their holding tanks. Sharks were then transported (∼20 min by vehicle) in insulated coolers filled with continuously aerated seawater back to the Centre de Recherche Insulaire et Observatoire de l'Environnement (CRIOBE) research station. At CRIOBE, sharks were maintained in flow-through aquaria (four sharks per 1000 l circular polyethylene tank, 168 cm diameter, 45 cm deep) so that ‘umbilical scar’ healing could be monitored in a controlled environment. After sharks had habituated to laboratory conditions and were eating regularly (5% of their body mass every other day, fresh black tuna), individuals were periodically retrieved from holding tanks using two mesh hand nets so that they could be re-measured and/or photographed to assess the progress of ‘umbilical scar’ healing. This sampling and measurement process took, on average, 4 min, after which time individuals were returned to their original holding tanks. Photographs of umbilical scars were taken at the beginning of the project, which commenced no sooner than 1 week after sharks had been captured and were feeding regularly in the laboratory setting (referred to as day 0) and then again periodically to monitor healing rates (days 6, 12 and 18). Photographs were imported into the open-source image processing software FIJI by ImageJ (version 2.0.0-rc-23/1.49m; Schindelin *et al*., 2012), scales were set to 1 cm using the ruler that was photographed with each shark, and then scars were traced using the freehand selection tool. Area (in square centimetres), perimeter (in centimetres) and shape descriptor measurement tools, including circularity [4π*area*(perimeter^2^)^−1^], aspect ratio (majoraxis*minoraxis^−1^) and roundness [4*area*(π*majoraxis^2^)], were used for each traced ‘umbilical scar’. Data were then compiled and compared over time using Student's paired *t*-tests for body measurements (comparing day 0 and day 24 only) and repeated-measures ANOVAs for scar dimensions (Systat Software, Inc., Chicago, IL, USA). All sharks were released back into their natal lagoons after 30 days in captivity.

### Monitoring injuries in adult sharks in Moorea

Also in Moorea, 241 free-swimming juvenile and adult blacktip reef sharks were photographed during 190 underwater surveys conducted between 2008 and 2010 by the CRIOBE laboratory in Moorea (Mourier *et al.*, 2012). Some of these animals were also observed when captured for genetic samples (Mourier and Planes, 2013). We used specific dorsal fin coloration patterns for photo-identification during underwater surveys (Mourier *et al.*, 2012). On occasion, individuals were observed with wounds or injuries due to mating, aggression or, potentially, stemming from anthropogenic causes. Injuries were photographed, and when possible, wound healing was monitored over time using subsequent photographs.

### Healing and survival of tagged sharks in Australia

**Figure 1:**
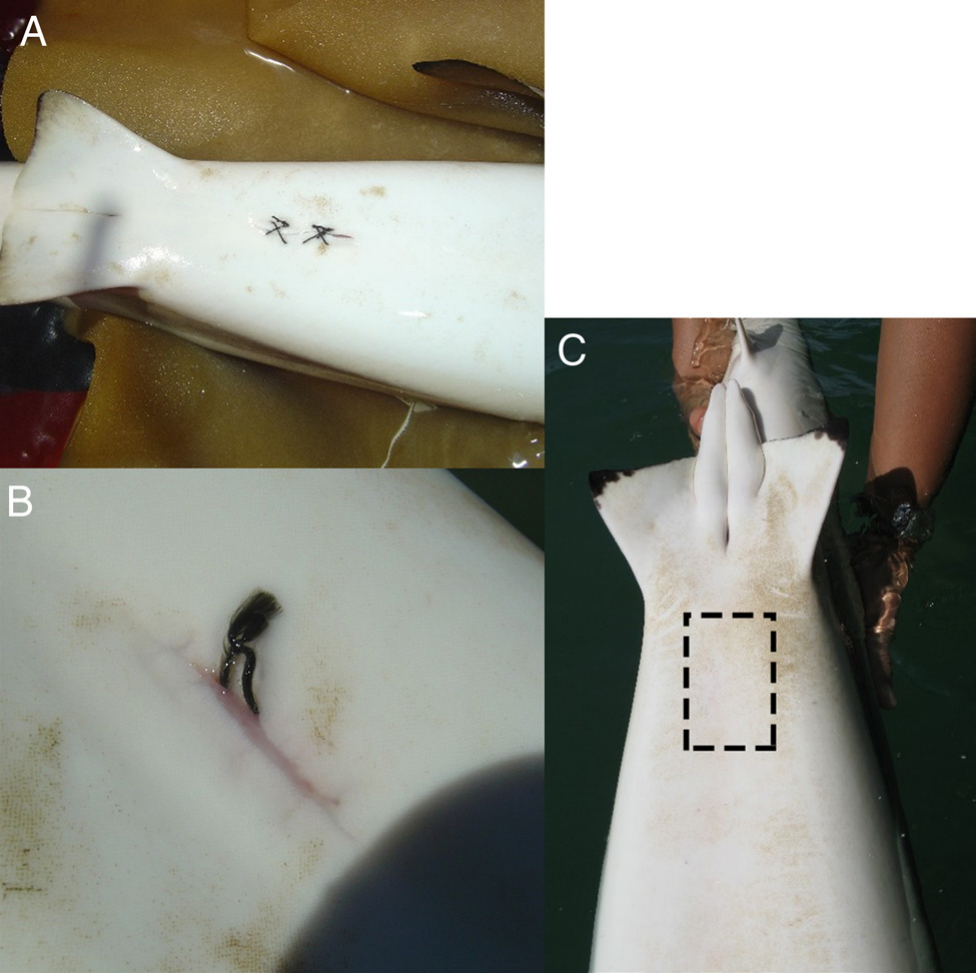
(**A**) Small incisions from the tagging procedure were closed using a cruciate suture pattern. (**B**) Recaptured animals showed advanced wound healing within 29 days. (**C**) Wound and scar at the incision point (dashed box) were undetectable within 179 days.

In Australia, we tagged 120 blacktip reef sharks in their first dorsal fins with Superflexitags (Dalton ID Systems, Henley-on-Thames, UK) as part of a long-term tag-recapture study (Chin *et al.*, 2013c). Of these animals, 27 sharks (ranging from 65 to 155 cm stretched total length) were also tagged with Vemco V16 acoustic tags (Vemco Ltd, AMIRIX Systems Inc., Halifax, Nova Scotia, Canada). Sharks were carefully restrained in foam cradles and inverted to induce tonic immobility. Acoustic tags were implanted into the abdominal cavity through a 3–4 cm incision made on the ventral side along the body midline. The incision was then closed with veterinary sutures (Model CT1; Ethicon Inc., Somerville, NJ, USA) using four cruciate-pattern sutures with two stiches each in the muscle and dermis layers (Fig. 1). All tagging procedures were carried out by trained personnel following strict protocols developed by veterinarians and approved by animal research ethics committees. The entire procedure for each shark was typically completed in <10 min. Sharks were then returned to their normal swimming position, closely monitored to ensure recovery, and then released. Long-term recovery and survival of these animals was directly recorded from field notes and photographs of individuals recaptured during subsequent capture–mark–recapture sampling, and inferred from long-term telemetry data (Chin *et al.*, 2013c).

## Results

### Umbilical scars of neonatal sharks in Moorea

**Figure 2:**
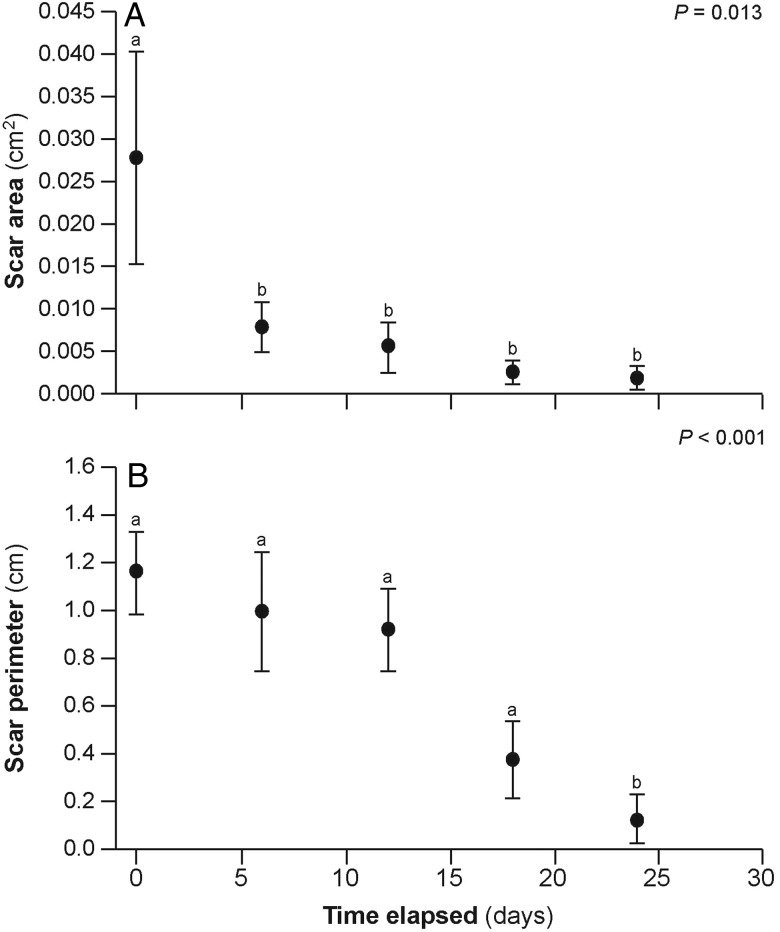
Temporal evolution of umbilical scar healing rate for both significant variables, scar area (**A**) and scar perimeter (**B**). See Table 1 for further details. Different letters demarcate statistically significant differences, and *P*-values are indicated in the top corner of each panel.

**Figure 3:**
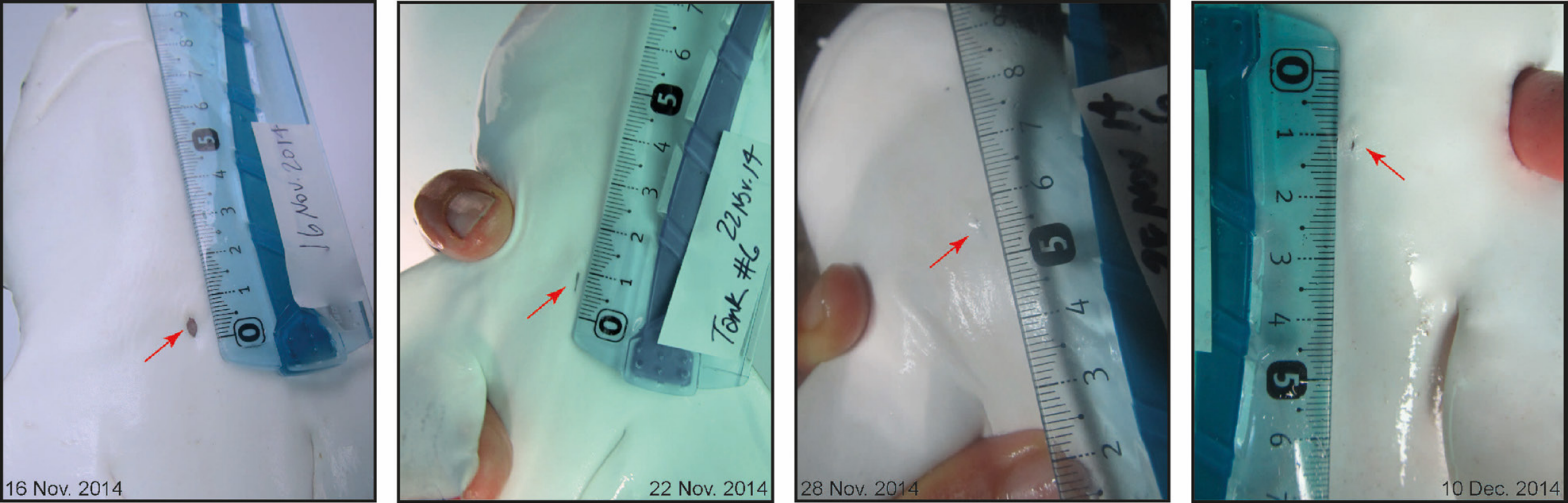
Representative photographs documenting the monitored healing rate of an individual neonatal blacktip reef shark showing the steps from an open scar on 16 November 2014 to a closed and almost completely healed scar on 10 December 2014.

Over the 24 day observation period, neonatal sharks significantly increased fork length by an average of 1.6% [*t*(11) = −2.83, *P* = 0.043; Table 1]. No significant differences were detected between 0 and 24 days for total length, body mass or body condition, even though there was a trend toward an increase in mass (Table 1). Scar dimensions changed over time. Scar area and scar perimeter were both significantly correlated with time, decreasing by 94 and 89%, respectively, over the 24 day period (*r* = −0.5080, *P* = 0.0113 and *r* = −0.6430, *P* = 0.0007, respectively; Table 1 and Fig. 2). Scar area decreased significantly within the first 6 days that sharks were being observed, after which there were no significant changes for the rest of the observation period (Fig. 2). Scar perimeter did not decrease significantly until toward the end of the observation period (Fig. 2). Interestingly, SEM also decreased over time, especially for scar area (Table 1 and Fig. 2). No significant correlations with time were determined for scar circularity, aspect ratio or roundness (Table 1). Representative photographs show that scars were initially open and round (day 0), then started to close and appeared more slit-like and, ultimately, toward the end of the observation period, scars appeared to be small pinholes in the skin (Fig. 3).

**Table 1:** Measurements of umbilical scars and body size of neonatal blacktip reef sharks to track healing

Time elasped (days)	Shark body measurements and calculations					Scar measurements and calculations				
	FL (cm)	TL (cm)	*B*_m_ (g)	Condition *B*_m_ × (FL^3^)^−1^ × 100	Condition *B*_m_ × (TL^3^)^−1^ × 100	Area (cm)	Perimeter (cm)	Circularity	Aspect ratio	Roundness
0	47.00 ± 1.05	59.50 ± 1.16	962.50 ± 76.10	0.92 ± 0.03	0.45 ± 0.02	0.0278 ± 0.0123	1.1643 ± 0.1724	0.2903 ± 0.1079	9.0370 ± 2.9684	0.2233 ± 0.0894
6						0.0081 ± 0.0030	0.9989 ± 0.2472	0.1799 ± 0.0935	9.9269 ± 2.2053	0.1544 ± 0.0671
12						0.0058 ± 0.0029	0.9249 ± 0.1667	0.1027 ± 0.0517	21.7497 ± 8.7327	0.1033 ± 0.0348
18						0.0028 ± 0.0014	0.3827 ± 0.1629	0.0180 ± 0.0140	2.8724 ± 1.1238	0.1558 ± 0.0610
24	47.63 ± 1.09*	59.63 ± 1.23	1068.75 ± 102.49	0.97 ± 0.04	0.49 ± 0.03	0.0018 ± 0.0014	0.1294 ± 0.1002	0.1479 ± 0.1146	1.0477 ± 0.8116	0.1061 ± 0.0822
Correlation						−0.5080	−0.6430	−0.3500	−0.1530	−0.2480
*P*-value						0.0113	0.0007	0.0939	0.4740	0.2420
Significance						*	***	NS	NS	NS

Statistical significance is indicated by asterisks (* for *P* < 0.05, ** for *P* < 0.01, and *** for *P* < 0.001). Abbreviation: NS, no significance.

### Monitoring injuries in adult sharks in Moorea

**Figure 4:**
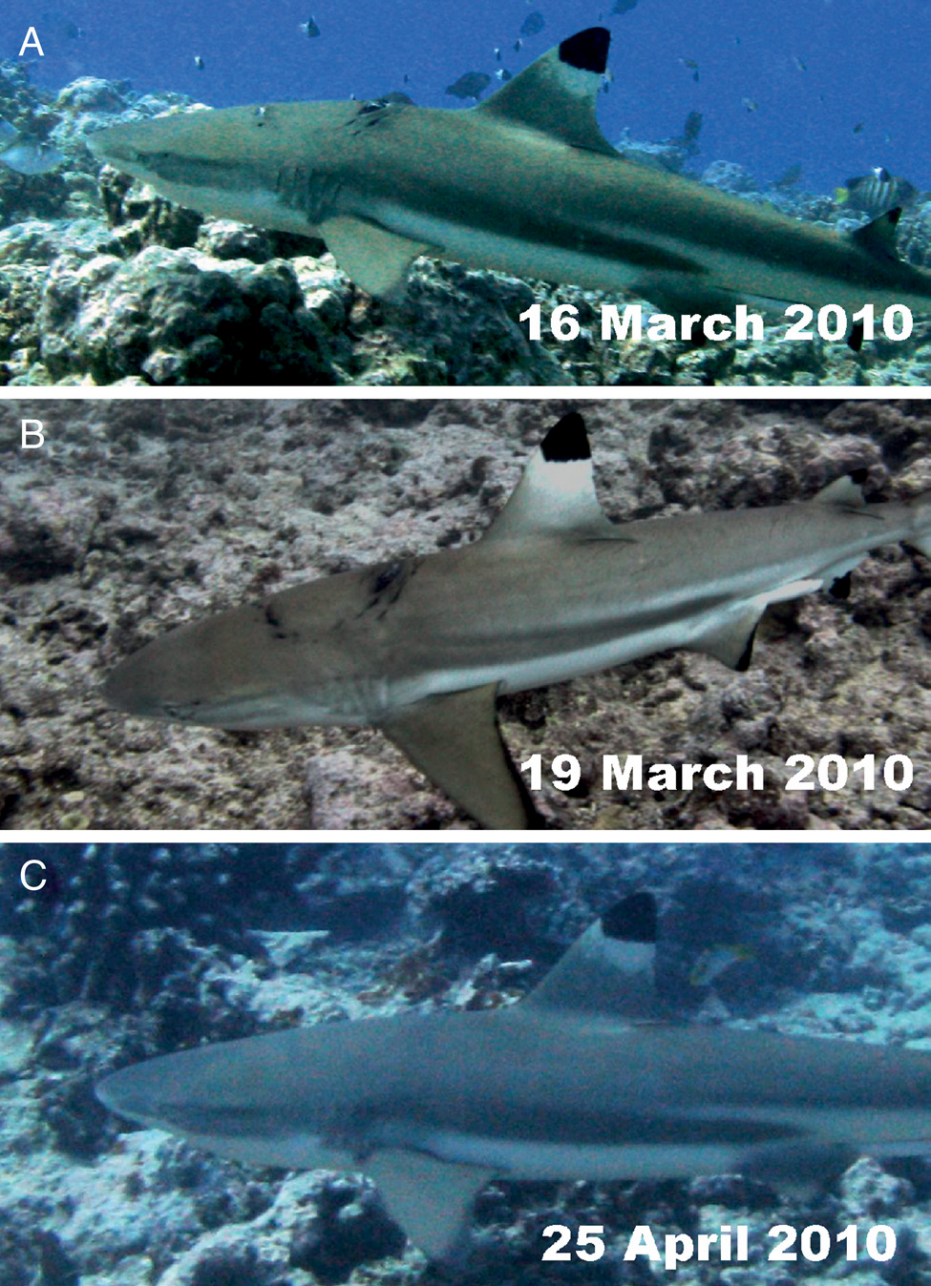
(**A**) Bite wound inflicted to a male blacktip reef shark by another shark species during an aggression or attempted predation. The wound closed within 3 days (**B**) and healed completely within 40 days (**C**).

**Figure 5:**
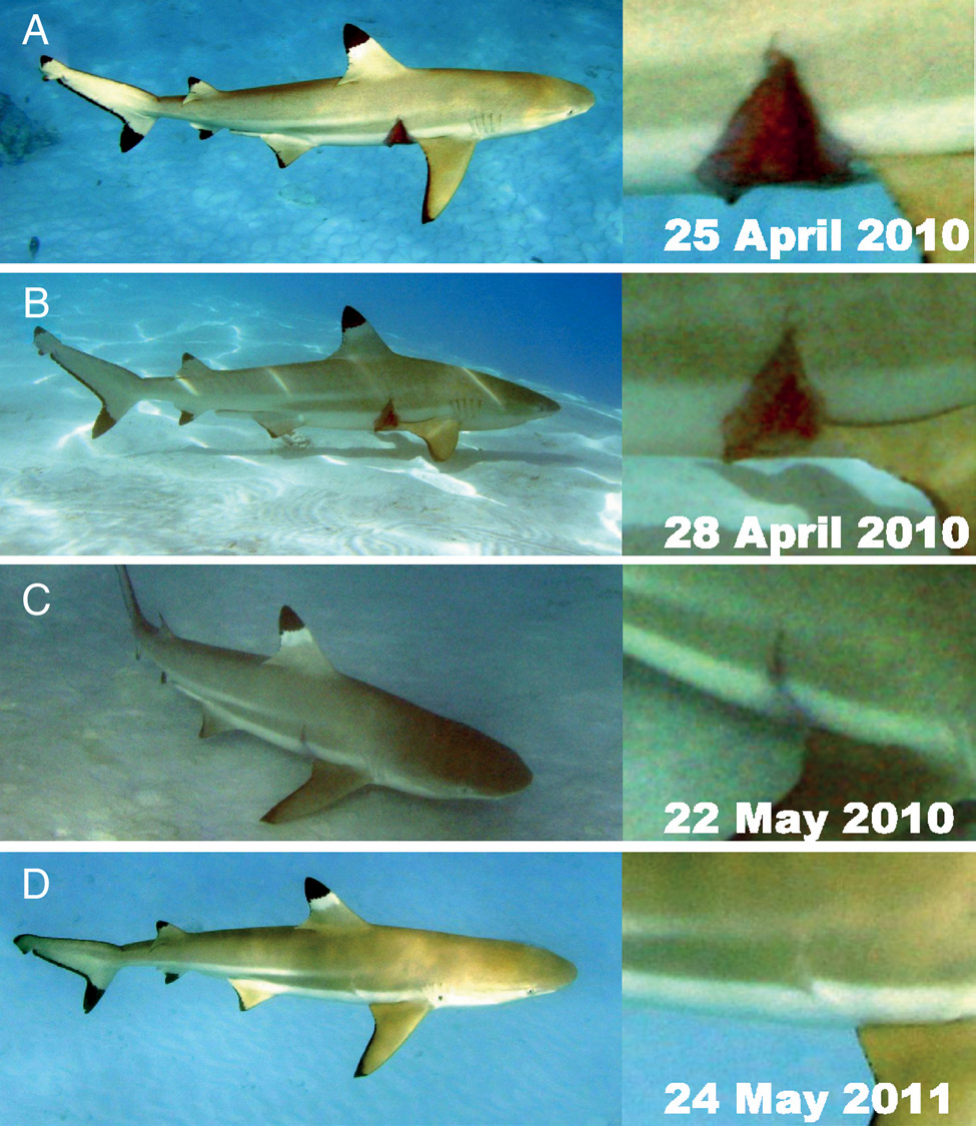
Healing of a deep wound from a suspected boat strike. (**A**) The bleeding wound was approximately 3–5 cm deep and 25 cm across. The wound stopped bleeding but remained open after 3 days (**B**), but fully closed within 27 days (**C**). (**D**) The scar had almost disappeared when the shark was resighted 13 months later. Note that photographs on the right are enlarged portions of original photographs and show details of the injury and healing.

In Moorea, 241 individual sharks were identified using photo-identification, and 193 were resighted (Mourier *et al.*, 2012). Sharks exhibited a range of injuries. A mature male was observed with a large, deep bite wound on the top of the head that was estimated to be 20 cm wide (Fig. 4). Owing to the large size of the wound, the injury was attributed to interspecific aggression or attempted predation, possibly by a grey reef or sicklefin lemon shark, both of which are common in the area. The wound was almost closed within 3 days and completely recovered within 40 days (Fig. 4). Mating scars on females also healed within a month (online supplementary materialFig. S1). A second male was observed with a large, bleeding, open wound on its right side that reached up to 25 cm in diameter and 3–5 cm in depth (Fig. 5). Given the high boat traffic in this section of the lagoon and that shark provisioning occurs at this site, the wound may have resulted from a boat strike. The shark was observed 3 days later, and the wound was still open but no longer bleeding. The shark was resighted 27 days later, showing complete closure of the wound (Fig. 5). One year later, the shark was observed, and the scar was barely visible (Fig. 5). This shark has recently (July 2015) been observed in the same area with no sign of previous injury. In other cases, sharks showed recovery from a fin injury (i.e. fin was split into two parts), and others demonstrated long-term survival after complete removal of the dorsal fin (online supplementary material Figs S2 and S3).

### Healing and survival of internally tagged sharks in Australia

**Figure 6:**
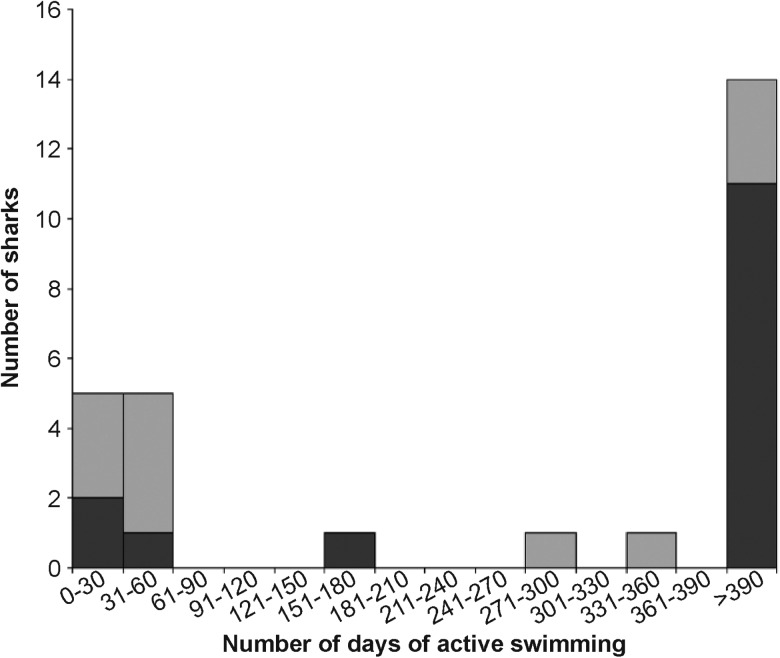
All 27 sharks were actively swimming after the acoustic tagging procedure, and 82% of the sharks were detected for months to years afterwards, suggesting long-term survival of these animals. Light grey indicates juveniles (<100 cm stretched total length), whereas dark grey indicates adults (>100 cm stretched total length).

In Australia, blacktip reef sharks appeared to recover rapidly from the acoustic tagging procedure, and all 27 animals were recorded as ‘actively swimming’ after release. In the days immediately after the procedure, all animals were detected on multiple receivers over a period of several days, suggesting that they were continuing to swim actively around the study site. The majority (*n* = 22) of tagged sharks were subsequently detected within the study area for periods of months to years (Fig. 6), suggesting long-term survival and residency. Four acoustically tagged sharks were recaptured and physically inspected 29, 46, 69 and 179 days after tagging. Photographs revealed that the incision was completely closed and the skin almost completely healed within 29 days of the procedure. By 129 days, there was no mark or scar visible (Fig. 1). Only five of the 27 tagged sharks were detected for fewer than 30 days (Fig. 6), and telemetry data suggested that they might either have left the study area, because the last detections were at the edge of the receiver array (*n* = 3), or were juveniles subject to predation (*n* = 2), as indicated by an abrupt change in swimming speed and movement patterns (A. Chin, unpublished data; Chin *et al.*, 2013a).

## Discussion

A range of external wounds across life-history stages in blacktip reef sharks were recorded from observations in French Polynesia and Australia, and data suggest that this species has a high capacity to recover from wounds and survive injuries. Small umbilical wounds in neonates decreased in surface area by 71% in less than a week and were barely detectable after 24 days. Wounds up to several centimetres long in juveniles and adults started to close within days and were undetectable within weeks to months, similar to healing rates observed in captive sharks (Reif, 1978). Fresh bite wounds in blacktip reef sharks in Moorea had completely healed within 3–5 weeks. This information can be important to infer date of birth for neonatal sharks based on the healing status of their umbilical scar (Aubrey and Snelson, 2007), which can also be helpful in other areas of research, such as determining key nursery areas (McCallister *et al.*, 2013), the timing of first dispersal (Chin *et al.* 2013a) or thresholds for trophic shifts from energy allocation from the mother to feeding autonomy (Matich *et al.* 2015). Healing rates in adults can provide a baseline that can be used to validate estimates of the timing of mating, because some studies use mating scars to indicate mating activity (Porcher 2005; Chin 2013b; Mourier and Planes 2013). Information about healing rates could also provide a coarse estimate of the timing of an initial injury, which could potentially assist fisheries and marine park compliance and management activities.

Healing may be especially important during early life stages. An open umbilical scar is a potential source of infection, which may be especially problematic in sensitive young sharks with newly developing immune systems. Once abandoned by their mothers, neonatal sharks have to be autonomous and manage energy allocation to different tasks, such as foraging (Matich *et al.* 2015), avoidance of predation, and exploration of diverse habitats with multiple environmental conditions (Chin *et al.* 2012, 2013a), which may also increase their exposure to pathogens. Indeed, nursery habitats are known for certain characteristics, including shallow, warm, hypoxic, but highly productive waters, characteristics that may heighten the risk of infection. Neonates already have to manage changes in their local environment and allocate resources and energy effectively in order to optimize growth and maximize survival; therefore, a decreased risk of infection owing to rapid healing of umbilical wounds would presumably be advantageous. This is an area requiring more research.

Sharks also recovered rapidly from internal acoustic tagging procedures and showed long-term (months to years) survival after release. Rapid healing of these minor injuries is, perhaps, not surprising given that the present study showed that blacktip reef sharks recovered completely from much larger wounds, such as deep, bleeding lacerations, and exhibited long-term survival after removal of the entire dorsal fin. The observed recovery and survival after injuries that include major trauma suggest that the chondrichthyan immune system must be effective at preventing infection (for review, see Luer *et al.*, 2004). These observations also provide some indication that, in some cases, individuals may be able to survive the loss of an entire fin. Nevertheless, the longer-term effects of this type of injury on shark health and fitness remain unknown.

A shark's resilience to physical injury suggests that post-release fishing mortality may depend more on the physiological stress response and handling practices (Skomal and Bernal, 2010; Eddy *et al*. 2016) than the physical injuries incurred during capture and release. However, it is important to note that, although sharks may be resilient to external injuries, such as superficial hooking, ingestion of hooks into the oesophagus or gastrointestinal tract can cause extensive internal damage, infection and/or inflammation that may significantly reduce individual fitness and post-release survival (Borucinska *et al.*, 2002). Consequently, care must be taken to minimize the likelihood of hook ingestion and stress in shark research and, as such, circle hooks are recommended over other types (Cooke *et al.*, 2012; Danylchuk *et al.*, 2014). Blacktip reef sharks appear to be robust to capture and handling as shown by low or absent mortality after tagging experiments (Fig. 6) and multiple recaptures in long-term monitoring studies (Chin *et al.* 2013c; Mourier *et al.*, 2013a). However, future work should investigate the level of stress following capture in blacktip reef sharks to determine directly the contribution of fishing practices on blacktip reef shark stress, health and mortality. This could be investigated through standardized capture-and-release experiments and measurements of blood chemistry, such as glucose and lactate concentrations (Danylchuk *et al*., 2014; Gallagher *et al.*, 2014). Preliminary work in Moorea has attempted to measure plasma cortisol as an indicator of stress using a cortisol enzyme immunoassay kit, but validation for *C. melanopterus* was unsuccessful owing to low concentration detectability (Mills *et al*., 2010). Ongoing studies are attempting to address this knowledge gap in blacktip reef sharks.

The present study also suggests that trained personnel following appropriate procedures can implant acoustic tags internally without compromising the long-term health and survival of the tagged animal. The physiological and immunological effects of conventional external tags on sharks have been examined directly in a few species, and studies suggest that tagging is unlikely to reduce fitness or increase mortality (Heupel and Bennett, 1997; Heupel *et al.*, 1998). However, there are few data available on survival and recovery after the surgical procedures used to implant internal telemetry tags. This is relevant, given the increase in use of telemetry tags in elasmobranch research (Heupel and Webber, 2012) and debates regarding the health and welfare of sharks during research activities. Indeed, this is a topical issue, with authors presenting varying views regarding research impacts and conservation benefits (Heupel and Simpfendorfer, 2010; Hammerschlag and Sulikowski, 2011). Concerns about potential impacts of internal tagging procedures have resulted in some studies using externally mounted tags fixed to the dorsal fin, even when this can compromise the amount of data available to inform conservation efforts of critically endangered species (C. Simpfendorfer, personal communication). External tags are often shed (for review, see Kohler and Turner, 2001) and may also become fouled by marine organisms, thus reducing data quality and, potentially, causing public concern when tagged animals are sighted. Given the observed rapid wound recovery and high survival from a range of injuries, including internal tagging procedures, we suggest that the benefits of internal tags are likely to outweigh the risks associated with the procedure.

Although we have documented rapid wound healing in blacktip reef sharks, recovery rates may differ between species, locations or injury types. The process that occurs during umbilical wound healing in neonates may be functionally and physiologically different from the healing process that occurs after physical injury. Furthermore, much longer injury healing times than we observed have been recorded in other species. Bansemer and Bennett (2010) observed hook injuries in grey nurse sharks (*Carcharias taurus*) and, although sharks appeared to survive these injuries, the resulting tissue necrosis took >6 months to heal. Differences in healing rates may also be due to environmental factors. Wound healing could be reduced in cooler waters owing to reduced metabolic rates, as evident in grey nurse sharks (Bansemer and Bennett, 2010), and may also explain the case in white sharks (*Carcharodon carcharias*) at Guadalupe Island (∼18–20°C), where ‘minor lacerations and abrasions’ were visible for several months (Domeier and Nasby-Lucas, 2007). Meanwhile, sicklefin lemon sharks in tropical waters exhibit similar healing rates to blacktip reef sharks (Buray *et al.*, 2009), providing further circumstantial evidence of faster healing rates in warmer waters. While interspecific variation, individual immunology or environmental factors may affect wound recovery, the relative importance of these variables for wound healing in sharks is yet to be explored.

Another unresolved factor is the range and extent of sublethal effects that could arise from injuries. Although our study provides information on individual long-term survival, the health and fitness of recaptured animals was not quantified, nor were any potential changes in ecology or behaviour. Presumably, additional energy would be allocated toward healing processes, and this energy would normally be allocated toward growth, feeding, swimming and other aspects of the animals' aerobic metabolic scope. Researchers are encouraged to document injuries and record healing rates on captive sharks in the laboratory and in field studies where possible so that we can begin to address some of these knowledge gaps.

In summary, this study suggests that elasmobranchs may be resilient to injuries, showing rapid healing from minor wounds and long-term survival from even major mechanical injuries. These are positive findings for elasmobranch conservation, especially considering that up to a quarter of all shark and ray species worldwide are threatened with extinction (Dulvy *et al*., 2014). Such findings also provide opportunities for management agencies and fisheries to increase post-release survival in elasmobranchs. For example, education programmes for recreational anglers could focus on encouraging minimal handling time and stress when releasing sharks, which could include cutting a line near the hook instead of repeatedly attempting to remove the hook. Anglers should also be made aware that sharks can recover from mechanical injury; therefore, sharks should be released even if the animal sustains injuries during the capture process. In commercial fisheries, shark bycatch is a prime issue. Management policies should acknowledge the resilience of elasmobranchs to mechanical injury and include this information in handling guides to justify early release of bycatch shark species. Instead of trying to retrieve the animal to remove a hook physically, fisheries managers could recommend that wire leaders are replaced with monofilament leaders so that handling and capture stress can be minimized. Circle hooks, especially those that will corrode, should also be used for these reasons. Managing agencies and research ethic committees should also consider the resilience of these animals to injuries when balancing the risks of proposed research against potential conservation outcomes. Although more data are needed regarding the various factors that influence healing rates and the long-term effects of injuries on individual health and fitness, all researchers and fishers involved in elasmobranch tagging programmes should be encouraged to document injuries and post-release survival rates whenever possible. This increased understanding about how sharks tolerate wounds and injuries and the effects on long-term survival will be invaluable to refining bycatch mitigation and handling practices in the future.

## Supplementary material

Supplementary material is available at *Conservation Physiology* online.

## Funding

The laboratory studies in Moorea were funded in part by the Institut des Récifs Coralliens du Pacifique (IRCP, Fellowship to J.L.R.) and the Australian Research Council Centre of Excellence for Coral Reef Studies, and the field studies were financially supported by the Direction à l′Environnement (DIREN) of French Polynesia, Coordination Unit of the Coral Reef Initiatives for the Pacific (CRISP Program) and Proscience. The Australian study was funded by the Australian Government MTSRF Program 4.8.4 (A.C.).

## Supplementary Material

Supplementary Data

## References

[COV062C1] Aubrey CW, Snelson FF Jr (2007) Early life history of the spinner shark in a Florida nursery. In McCandless CT, Kohler NE, Pratt HL Jr, eds, Shark Nursery Grounds of the Gulf of Mexico and the East Coast Waters of the United States. American Fisheries Society, Symposium 50, Bethesda, MD, pp 175–179.

[COV062C2] Bansemer CS, Bennett MB (2010) Retained fishing gear and associated injuries in the east Australian grey nurse sharks (*Carcharias taurus*): implications for population recovery. Mar Freshwater Res 61: 97–103.

[COV062C3] Borucinska J, Kohler N, Natanson L, Skomal G (2002) Pathology associated with retained fishing hooks in blue sharks, *Prionace glauca* (L.), with implications for their conservation. J Fish Dis 25: 515–521.

[COV062C4] Buray N, Mourier J, Planes S, Clua E (2009) Underwater photo-identification of sicklefin lemon sharks, *Negaprion acutidens*, at Moorea (French Polynesia). Cybium 33: 21–27.

[COV062C5] Carrier JC, Pratt HL, Martin LK (1994) Group reproductive behaviors in free-living nurse sharks, *Ginglymostoma cirratum*. Copeia 1994: 646–656.

[COV062C200] Chin A, Tobin AJ, Simpfendorfer CA, Heupel MR (2012) Reef sharks and inshore habitats: patterns of occurrence and implications for vulnerability. Mar Ecol Prog Ser 460: 115–125.

[COV062C6] Chin A, Heupel MR, Simpfendorfer CA, Tobin AJ (2013a) Ontogenetic movements of juvenile blacktip reef sharks: evidence of dispersal and connectivity between coastal habitats and coral reefs. Aquat Conserv 23: 468–474.

[COV062C7] Chin A, Simpfendorfer C, Tobin A, Heupel M (2013b) Validated age, growth and reproductive biology of *Carcharhinus melanopterus*, a widely distributed and exploited reef shark. Mar Freshwater Res 64: 965–975.

[COV062C8] Chin A, Tobin AJ, Heupel MR, Simpfendorfer CA (2013c) Population structure and residency patterns of the blacktip reef shark *Carcharhinus melanopterus* in turbid coastal environments. J Fish Biol 82: 1192–1210. 23557299 10.1111/jfb.12057

[COV062C9] Cooke SJ, Nguyen VM, Murchie KM, Danylchuk AJ, Suski CD (2012) Scientific and stakeholder perspectives on the use of circle hooks in recreational fisheries. Bull Mar Sci 88: 395–410.

[COV062C10] Danylchuk AJ, Suski C, Mandelman JW, Murchie KJ, Haak CR, Brooks AM, Cooke SJ (2014) Hooking injury, physiological status and short-term mortality of juvenile lemon sharks (*Negaprion bevirostris*) following catch-and-release recreational angling. Conserv Physiol 2: doi:10.1093/conphys/cot036. 10.1093/conphys/cot036 PMC4732486 27293620

[COV062C11] Domeier ML, Nasby-Lucas N (2007) Annual re-sightings of photographically identified white sharks (*Carcharodon carcharias*) at an eastern Pacific aggregation site (Guadalupe Island, Mexico). Mar Biol 150: 977–984.

[COV062C12] Dulvy NK, Fowler SL, Musick JA, Cavanagh RD, Kyne PM, Harrison LR, Carlson JK, Davidson LN, Fordham SV, Francis MP et al (2014) Extinction risk and conservation of the world's sharks and rays. eLife 3: e00590. 24448405 10.7554/eLife.00590 PMC3897121

[COV062C13] Eddy C, Brill R, Bernal D (2016) Rates of at-vessel mortality and post-release survival of pelagic sharks captured with tuna purse seines around drifting fish aggregating devices (FADs) in the Equatorial Eastern Pacific Ocean. Fish Res 174: 109–117.

[COV062C14] Gallagher AJ, Serafy JE, Cooke SJ, Hammerschlag N (2014) Physiological stress response, reflex impairment, and survival of five sympatric shark species following experimental capture and release. Mar Ecol Prog Ser 496: 207–218.

[COV062C15] Hammerschlag N, Sulikowski J (2011) Killing for conservation: the need for alternatives to lethal sampling of apex predatory sharks. Endanger Species Res 14: 135–140.

[COV062C16] Heupel MR, Bennett MB (1997) Histology of dart tag insertion sites in the epaulette shark. J Fish Biol 50: 1034–1041.

[COV062C17] Heupel MR, Simpfendorfer CA (2010) Science or slaughter: need for lethal sampling of sharks. Conserv Biol 24: 1212–1218. 20337690 10.1111/j.1523-1739.2010.01491.x

[COV062C18] Heupel MR, Webber DM (2012) Trends in acoustic tracking: where are the fish going and how will we follow them? In McKenzie JR, Parsons B, Seitz AC, Kopf RK, Mesa MG, Phelps Q, eds, Advances in Fish Tagging and Marking Technology. American Fisheries Society, Symposium 50, Bethesda, MD, pp 219–231.

[COV062C19] Heupel MR, Simpfendorfer CA, Bennett MB (1998) Analysis of tissue responses to fin tagging in Australian carcharhinids. J Fish Biol 52: 610–620.

[COV062C20] Hoyos-Padilla M, Papastamatiou YP, O'Sullivan J, Lowe CG (2013) Observation of an attack by a cookiecutter shark (*Isistius brasiliensis*) on a white shark (*Carcharodon carcharias*). Pac Sci 67: 129–134.

[COV062C21] Kajiura SM, Sebastian AP, Tricas TC (2000) Dermal bite wounds as indicators of reproductive seasonality and behaviour in the Atlantic stingray, *Dasyatis sabina*. Environ Biol Fish 58: 23–31.

[COV062C22] Kohler NE, Turner PA (2001) Shark tagging: a review of conventional methods and studies. Environ Biol Fish 60: 191–223.

[COV062C23] Luer CA, Walsh CJ, Bodine AB (2004) The immune system of sharks, skates and rays. In Carrier JC, Musick JA, Heithaus HR, eds, Biology of Sharks and Their Relatives. CRC Press, Boca Raton, FL, pp 369–395.

[COV062C300] McCallister M, Ford R, Gelsleichter J (2013) Abundance and distribution of sharks in Northeast Florida waters and identification of potential nursery habitat. Mar Coast Fish 5: 200–210.

[COV062C24] McCauley DJ, Papastamatiou YP, Young HS (2010) An observation of mating in free-ranging blacktip reef sharks, *Carcharhinus melanopterus*. Pac Sci 64: 349–352.

[COV062C25] Matich P, Kiszka JJ, Heithaus MR, Mourier J, Planes S (2015) Short-term shifts of stable isotope (δ^13^C, δ^15^N) values in juvenile sharks within nursery areas suggest rapid shifts in energy pathways. J Exp Mar Biol Ecol 465: 83–91.

[COV062C26] Mills SC, Mourier J, Galzin R (2010) Plasma cortisol and 11-ketotestosterone enzyme immunoassay (EIA) kit validation for three fish species: the orange clownfish *Amphiprion percula*, the orangefin anemonefish *Amphiprion chrysopterus* and the blacktip reef shark *Carcharhinus melanopterus*. J Fish Biol 77: 769–777. 20701653 10.1111/j.1095-8649.2010.02693.x

[COV062C27] Mourier J, Planes S (2013) Direct genetic evidence for reproductive philopatry and associated fine-scale migrations in female blacktip reef sharks (*Carcharhinus melanopterus*) in French Polynesia. Mol Ecol 22: 201–214. 23130666 10.1111/mec.12103

[COV062C28] Mourier J, Vercelloni J, Planes S (2012) Evidence of social communities in a spatially structured network of a free-ranging shark species. Anim Behav 83: 389–401.

[COV062C29] Mourier J, Mills SC, Planes S (2013a) Population structure, spatial distribution and life-history traits of blacktip reef sharks *Carcharhinus melanopterus*. J Fish Biol 82: 979–993. 23464555 10.1111/jfb.12039

[COV062C30] Mourier J, Planes S, Buray N (2013b) Trophic interactions at the top of the coral reef food chain. Coral Reefs 32: 285–285.

[COV062C400] Papastamatiou YP, Lowe CG, Caselle JE, Friedlander AM (2009) Scale-dependent effects of habitat on movements and path structure of reef sharks at a predator-dominated atoll. Ecology 90: 996–1008. 19449694 10.1890/08-0491.1

[COV062C31] Porcher IF (2005) On the gestation period of the blackfin reef shark, *Carcharhinus melanopterus*, in waters off Moorea, French Polynesia. Mar Biol 146: 1207–1211.

[COV062C32] Pratt HL (1979) Reproduction in the blue shark, *Prionace glauca*. Fish Bull 77: 445–470.

[COV062C33] Reif WE (1978) Wound healing in sharks – form and arrangement of repair scales. Zoomorphologie 90: 101–111.

[COV062C34] Riley MJ, Harman A, Rees RG (2009) Evidence of continued hunting of whale sharks *Rhincodon typus* in the Maldives. Environ Biol Fish 86: 371–374.

[COV062C35] Schindelin J, Arganda-Carreras I, Frise E, Kaynig V, Longair M, Pietzsch T, Preibisch S, Rueden C, Saalfeld S, Schmid B et al (2012) Fiji: an open-source platform for biological-image analysis. Nat Methods 9: 676–682. 22743772 10.1038/nmeth.2019 PMC3855844

[COV062C36] Skomal GB, Bernal D (2010) Physiological responses to stress. In Carrier JC, Musick JA, Heithaus MR, eds, Sharks and Their Relatives II: Biodiversity, Adaptive Physiology and Conservation. CRC Press, Boca Raton, FL, pp 459–490.

[COV062C37] Towner A, Smale MJ, Jewell O (2012) Boat strike wound healing in *Carcharodon carcharias*. In Domeier ML, eds, Global Perspectives on the Biology and Life History of the White Shark. CRC Press, Boca Raton, FL, pp 77–84.

[COV062C38] Vignaud TM, Mourier J, Maynard JA, Leblois R, Spaet J, Clua E, Neglia V, Planes S (2014) Blacktip reef sharks, *Carcharhinus melanopterus*, have high genetic structure and varying demographic histories in their Indo-Pacific range. Mol Ecol 23: 5193–5207. 25251515 10.1111/mec.12936

[COV062C39] Walker TI (2005) Management measures. In Musick JA, Bonfil R, eds, Management Techniques for Elasmobranch Fisheries. FAO Fisheries Technical Paper No. 474. Food and Agricultural Organization, Rome, pp 285–321.

[COV062C40] Wegner NC, Cartamil DP (2012) Effects of prolonged entanglement in discarded fishing gear with substantive biofouling on the health and behavior of an adult shortfin mako shark, *Isurus oxyrinchus*. Mar Pollut Bull 64: 391–394. 22172235 10.1016/j.marpolbul.2011.11.017

