# Supplementary material for: Blacktip reef sharks (Carcharhinus melanopterus) show high capacity for wound healing and recovery following injury
Source: Conserv Physiol. 2015 Dec 21;3(1):cov062. doi: 10.1093/conphys/cov062 (PMC4778477; doi:10.1093/conphys/cov062)

Supporting Information 1:

A female blacktip reef shark with recent mating scars (A) which started to heal after 8 days (B) and were almost completely healed after 15 days (C).


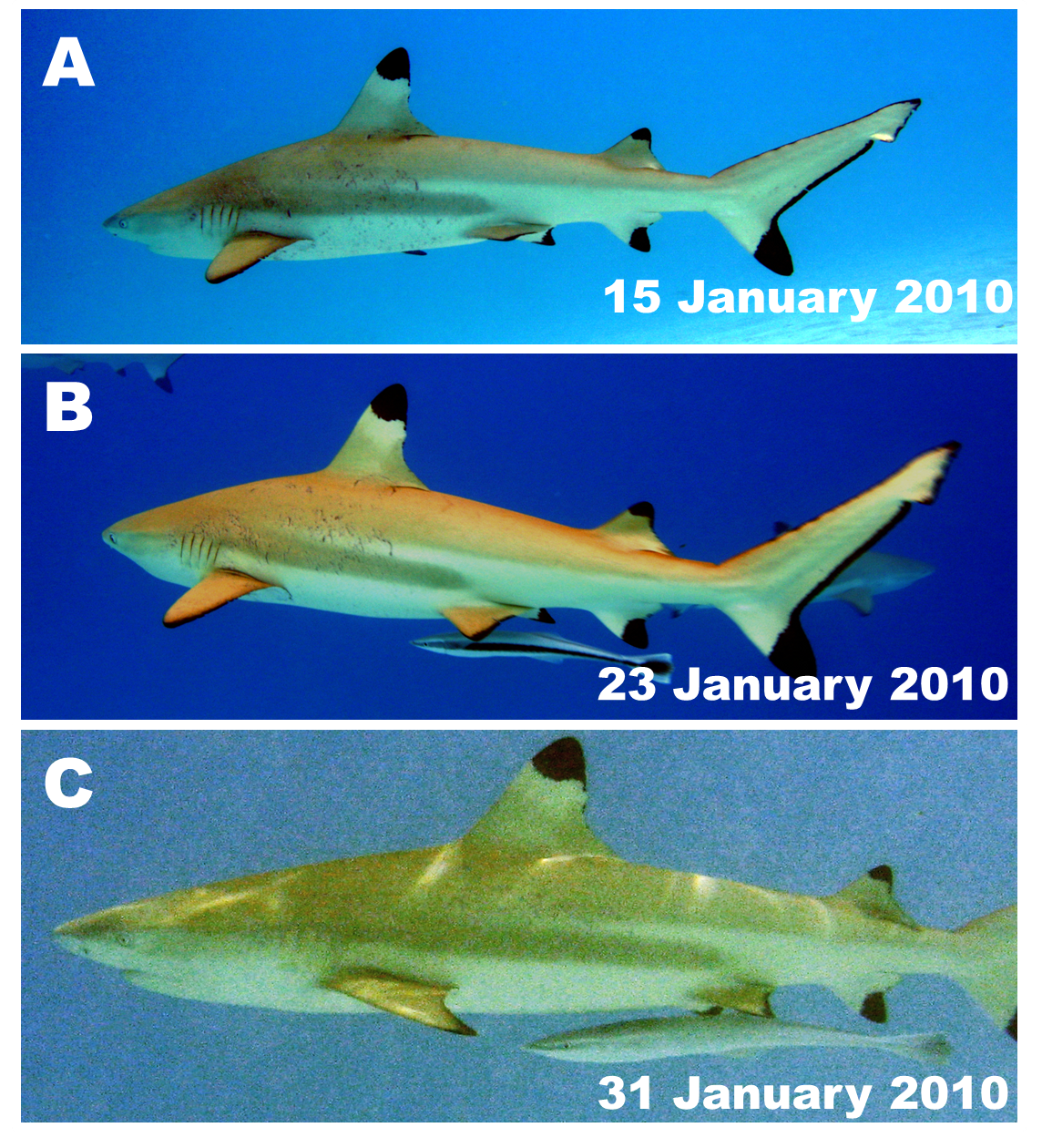


Supporting Information 2:

Progressive and rapid healing of a 10 cm vertical cut on the first dorsal fin of a male blacktip reef shark between April 2008 and May 2008. The two parts of the injured fin (A) merged back together within 33 days (B), with no visible wound or scar after 237 days (C).


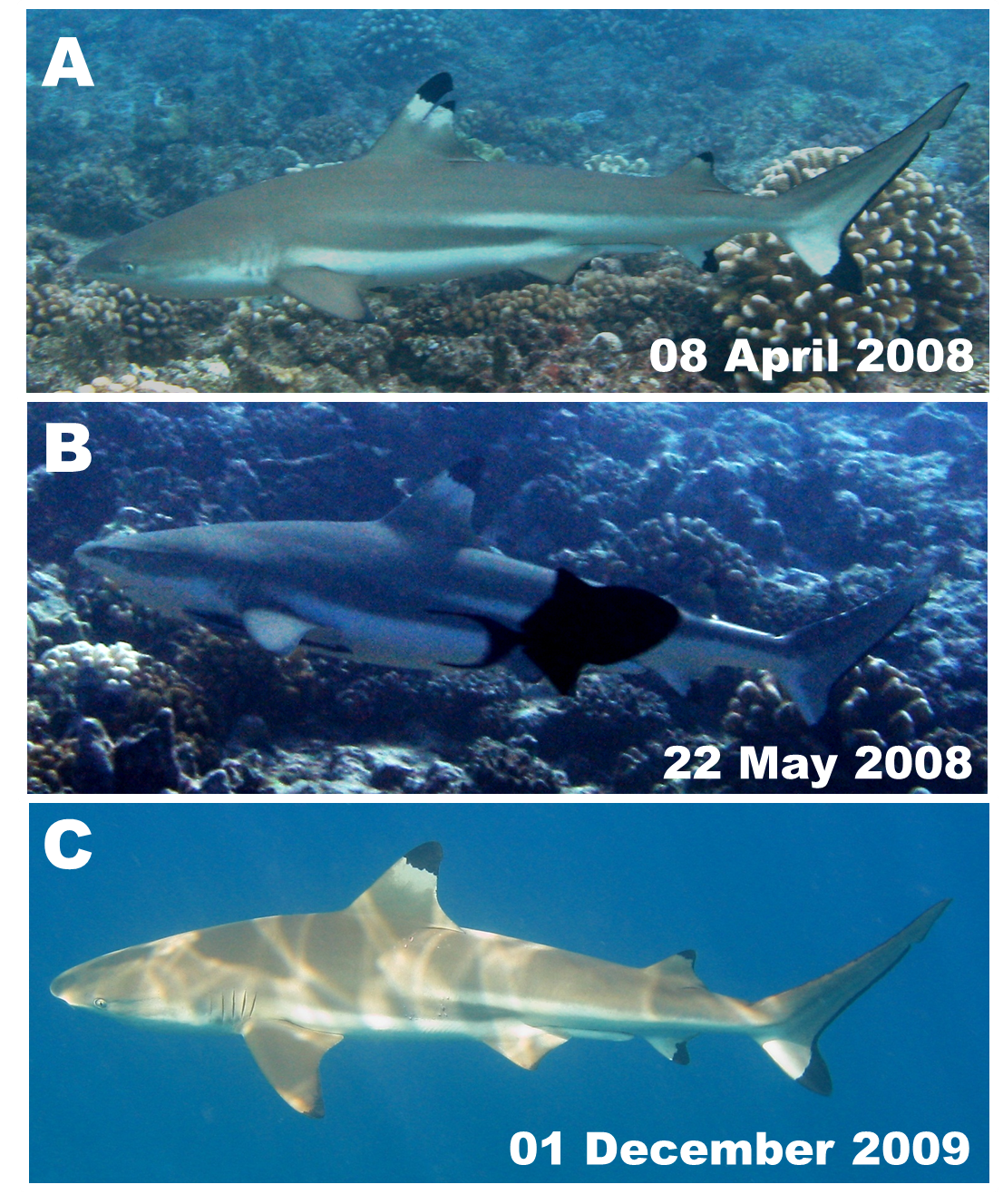


Supporting Information 3:

Observations of free-swimming blacktip reef sharks that survived removal of their first dorsal fin after a finning fishing procedure. (A) In 2008, a mature male was first observed swimming freely on the fore reef at Moorea without any first dorsal fin. The cut was completely healed and the shark was observed again in 2010 indicating long-term survival. (B) In 2012, a mature female was caught in the lagoon lacking the first dorsal fin. The injury completely healed and the female appeared in good health. (Photo credits: A-Johann Mourier; B-Martin Romain & Elodie Raimondi).


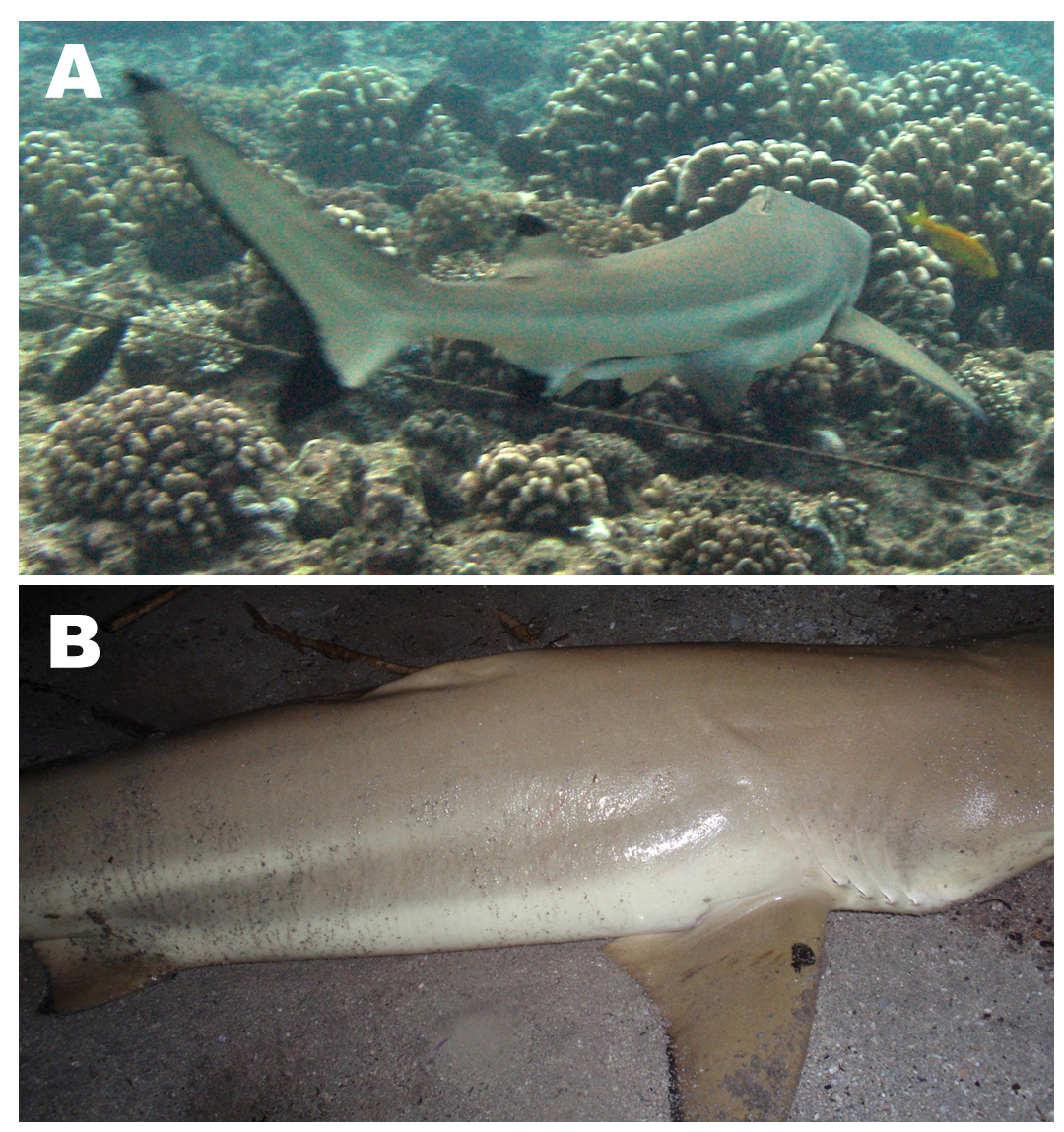

Supplement: Supplementary Data [file cov062supp.zip › cov062supp.docx]
